# COVID-19 mRNA vaccine in pregnancy and newborn passive immunization: a case report

**DOI:** 10.2144/fsoa-2021-0043

**Published:** 2021-10-29

**Authors:** Nadine El Kassis, Wael Abdallah, Rim Abou Chakra, Wissam Arab, Serge Sassine, David Atallah

**Affiliations:** ^1^Department of Gynecology & Obstetrics, Hôtel-Dieu de France University Hospital, Saint Joseph University, Beirut, 116-5137, Lebanon

**Keywords:** COVID-19, newborn immunization, pregnancy, vaccine

## Abstract

**Aim:** Evaluating the newborn passive immunization after maternal vaccination against SARS-COV-2. **Case presentation:** We present the case of a pregnant woman, with no prior history of COVID-19 infection, who got her second dose of mRNA vaccine against SARS-COV-2, 3 days before the start of her spontaneous labor. She was delivered by cesarean section after dynamical dystocia. Placental cord blood was retrieved immediately and sent to evaluate the titers of COVID-19 antibodies. Vaccine-generated antibodies were present in the umbilical cord with IgG spike >100 AU/ml. **Conclusion:** By reviewing the literature, vaccination seems to give hope about the potential protective effect of the maternal vaccination on her baby. Thus, pregnant women deserve a priority in the COVID-19 vaccination program.

## Lay abstract

Counseling pregnant patients about COVID-19 vaccine safety for her and her fetus is a common issue facing obstetricians. The WHO does not recommend the vaccination for pregnant women except for patients considered with unavoidable risk of high exposure. We present the case of a pregnant woman, with no prior history of COVID-19, who got her second dose of mRNA vaccine against SARS-COV-2, 3 days before the start of her spontaneous labor. Vaccine-generated antibodies were present in the umbilical cord. In conclusion, vaccination seems to give hope about the potential protective effect of the maternal vaccination on her baby.

Counseling pregnant patients about COVID-19 vaccine safety for her and her fetus is a common issue facing obstetricians. Some vaccines, like live vaccines, are restricted due to their harmful effect on the baby. On the other hand, inactivated seasonal flu vaccine and the whooping cough vaccine are recommended during pregnancy, according to the CDC [1]. In the setting of the high surge of COVID-19 infection in Lebanon, reaching up to 33% incidence based on the Lebanese ministry of public health daily data, there is an increased incidence of COVID-19 among pregnant women. The recent vaccines for COVID-19 have offered the population a new hope, but the vaccination is still debatable when it comes to pregnant women. However, pregnant women infected with SARS-COV-2 are at higher risk of severe illness than nonpregnant women [2]. Although pregnant patients were excluded from the clinical trials evaluating the vaccine’s safety, data on accidental pregnancies that happened during these trials showed that mRNA vaccine are safe during pregnancy [3]. The mRNA COVID-19 vaccine induces spike protein-specific neutralizing antibodies associated with protective immunity [4]. However, the WHO does not recommend the vaccination for pregnant women except for patients considered with unavoidable risk of high exposure, like health workers [5]. The immune transfer to neonates across the placenta among vaccinated pregnant women still presented insufficient data.

## Case presentation

We report a case of a 30-year old pregnant dentist (gravida 2, para 1) to evaluate the outcomes of maternal COVID-19 mRNA vaccination on her newborn. The patient underwent her pregnancy without any complications. She is considered a health worker at high risk of COVID-19 exposure and constantly tested negative for COVID-19 PCR with no history of COVID-19 infection. She got her first dose of COVID-19 mRNA (Pfizer-BioNTech) at 33^+3^ weeks and her second dose at 36^+3^ weeks. She presented to the delivery room at 36^+6^ weeks with spontaneous labor and a BISHOP score >6. The PCR COVID-19 (genes N and E) at the moment of admission, performed upon the hospital protocol’s request, returned negative. Maternal vaccine-induced antibody titers were >100 UI/ml (Abbott, IL, USA). The labor was long and she had dynamical dystocia during the first stage of labor. Thus, she was operated on a cesarean section after failure to progress. She delivered a baby girl with a fetal weight of 2640 g. Apgar score was 8 and 9 at 1 and 5 min respectively. Placental cord blood was retrieved immediately without maternal blood contamination; thereafter, and sent to evaluate the titers of COVID 19 antibodies. Quantitative measurement of anti-SARS-CoV-2 antibodies was used by automated chemiluminescent anti-SARS-COV-2 antibodies detecting S protein (Abbott). The titers of vaccine-generated antibodies were present in the umbilical cord with IgG spike >100 AU/ml. It is not possible in our case to determine the rate of transfer of antibodies from mother to fetus.

## Discussion & conclusion

This case aims to highlight the possibility of the transfer of vaccine-generated antibodies through the placenta to neonates. By reviewing the literature, we identified two articles concerning the placental transfer of vaccine-induced antibodies [6,7]. However, it is common to find vaccine-induced IgG in the fetal serum by a transfer across the placenta after tetanus, diphtheria, pertussis and flu vaccines [8,9]. Of concern, mRNA vaccination against SARS-COV-2 provides the formation of antibodies in maternal serum, but their placental transfer to infants is still unclear. The mRNA COVID-19 vaccine generates spike protein-specific antibodies and induces neutralizing anti-S IgG antibodies with associated protective immunity [4]. A prospective cohort study that was published in March 2021 concluded that vaccine-generated antibodies were found in all umbilical cord blood among ten vaccinated pregnant women [6]. We have a similar result in our case. Of concern, maternal IgG are able to cross the placenta and provide immunity to the newborn. By reviewing the literature, we found that neutralizing antibody titers were lower in the umbilical cord comparing with the maternal serum, without a statistically significant difference [6]. However, there is not enough data to identify the optimal time for vaccination to have the placental immune transfer. Even patients who took only the first dose of mRNA vaccination have presented IgG in the umbilical cords [6]. In our case, 3 days after the second dose of mRNA vaccination were enough to find efficient immunity in the newborn’s serum (>100 UI/ml). Thus, we can conclude that the maternal vaccination presents a potential infant’s protection. On the other hand, the duration of antibody in these newborns is not yet identified. In our case, the pediatrician suggested evaluating the infant antibodies titers after 3 and 6 months. The newborn was lost to follow up as the family emigrated after delivery. Further studies are needed to assess the importance of the follow up of infant antibodies titers and the optimal time of maternal vaccination. Furthermore, multiple reports have documented a vertical transmission of SARS-COV-2 to the fetus [10]. Thus, maternal vaccination could provide fetal protection against COVID 19, but there is no sufficient data to evaluate the longevity of antibodies in fetal serum. Additionally, we do not know if the spontaneous initiation of labor and the dynamical dystocia are associated with the vaccination done a couple of days ago. There are no data concerning the impact of vaccination on labor.

Finally, it is true that data are still lacking to help providers to advise pregnant women about the vaccination, but the review of the literature seems to give hope about the potential protective effect of the maternal vaccination on her baby. Thus, pregnant women deserve a priority in the COVID-19 vaccination program.

**Executive summary**

**Background**

- COVID-19 vaccine safety for pregnant women is a common issue facing obstetricians.
- By reviewing the literature, mRNA vaccine seems to be safe during pregnancy.
- The immune transfer to neonates across the placenta among vaccinated pregnant women still presented insufficient data.

**Case presentation**

- A pregnant woman, with no prior history of COVID-19 infection, got her second dose of mRNA vaccine against SARS-COV-2 and presented with spontaneous labor after 3 days.
- Vaccine-generated antibodies were present in the umbilical cord with IgG spike >100 AU/ml.

**Discussion & conclusion**

- The mRNA COVID-19 vaccine generates spike protein-specific antibodies and induces neutralizing anti-S IgG antibodies with associated protective immunity.
- Maternal IgG are able to cross the placenta and provide immunity to the newborn.
- Pregnant women deserve a priority in the COVID-19 vaccination program.
